# Chronic Low-Grade Inflammation in Childhood Obesity Is Associated with Decreased IL-10 Expression by Monocyte Subsets

**DOI:** 10.1371/journal.pone.0168610

**Published:** 2016-12-15

**Authors:** Rafael T. Mattos, Nayara I. Medeiros, Carlos A. Menezes, Rafaelle C. G. Fares, Eliza P. Franco, Walderez O. Dutra, Fabrício Rios-Santos, Rodrigo Correa-Oliveira, Juliana A. S. Gomes

**Affiliations:** 1 Laboratório de Biologia das Interações Celulares, Departamento de Morfologia, Instituto de Ciências Biológicas, Universidade Federal de Minas Gerais, Belo Horizonte, MG, Brasil; 2 Laboratório de Imunologia Celular e Molecular, Centro de Pesquisa René Rachou, FIOCRUZ, Belo Horizonte, MG, Brasil; 3 Departamento de Genética, Universidade Estadual de Santa Cruz, Ilhéus, BA, Brasil; 4 Serviço de Medicina Preventiva da Unimed, Aracaju, SE, Brasil; 5 Instituto Nacional de Ciência e Tecnologia em Doenças Topicais—INCT-DT; 6 Faculdade de Medicina, Departamento de Ciências Básicas da Saúde, Universidade Federal de Mato Grasso, Cuiabá, MT, Brasil; Institut Cochin, FRANCE

## Abstract

Chronic low-grade inflammation is related to the development of comorbidities and poor prognosis in obesity. Monocytes are main sources of cytokines and play a pivotal role in inflammation. We evaluated monocyte frequency, phenotype and cytokine profile of monocyte subsets, to determine their association with the pathogenesis of childhood obesity. Children with obesity were evaluated for biochemical and anthropometric parameters. Monocyte subsets were characterized by flow cytometry, considering cytokine production and activation/recognition molecules. Correlation analysis between clinical parameters and immunological data delineated the monocytes contribution for low-grade inflammation. We observed a higher frequency of non-classical monocytes in the childhood obesity group (CO) than normal-weight group (NW). All subsets displayed higher TLR4 expression in CO, but their recognition and antigen presentation functions seem to be diminished due to lower expression of CD40, CD80/86 and HLA-DR. All subsets showed a lower expression of IL-10 in CO and correlation analyses showed changes in IL-10 expression profile. The lower expression of IL-10 may be decisive for the maintenance of the low-grade inflammation status in CO, especially for alterations in non-classical monocytes profile. These cells may contribute to supporting inflammation and loss of regulation in the immune response of children with obesity.

## Introduction

Obesity is a multifactorial disease, characterized by an increase of body fat, that can be associated with metabolic changes [1, 2] and considered a serious public health problem with a large proportion and prevalence. World Health Organization (WHO) estimates 600 million of people with obesity worldwide, of which 42 million are children [3].

Recent studies have established association between obesity and systemic chronic low-grade inflammation [4, 5]. Moreover, it has been proposed that perturbation of inflammation is critically linked to nutrient metabolic pathways and obesity-associated complications, such as insulin resistance and type 2 diabetes [6–8]. The inflammatory effects of obesity may be systemic, causing metabolic disorders and altering cellular functions. Obesity-associated low-grade inflammation appears to originate primarily from macrophage infiltration in adipose tissue [9]. Thus, the interplay among monocytes/macrophages and inflammatory mediators can play a critical role in pathogenesis of obesity.

Human bloodstream monocytes are classified into three subsets, according to the expression patterns of the lipopolysaccharide (LPS) receptor, CD14, and the FcγIII receptor CD16, namely classical (CD14^++^CD16^-^), intermediate (CD14^++^CD16^+^) and non-classical (CD14^+^CD16^++^) [10]. These subsets differ significantly in proportion, phenotype and function [10].

Classical monocytes compose the majority of total monocytes. Functionally, they are phagocytes that originate the M1 macrophage and foam cells, which ingest native low-density lipoprotein (LDL), produce reactive oxygen species (ROS) and secret cytokines during immune response to infections or acute inflammatory process [10, 11]. Intermediate monocytes are a minor subset of total monocytes that shares phenotypic profile with classical and non-classical monocytes. This subset is very important as it characterizes the transition phenotype between monocytes subsets [11, 12]. Non-classical monocytes comprise nearly 10% of total monocyte count and they are wake phagocytes, but inflammatory cytokines producers, such as TNF-α and IL-1β [11]. This subset is the more efficient in antigen presentation, and is related to chronic inflammation disorders [13].

Recent studies have shown that monocytes contribute to inflammation and metabolic alterations [6, 8, 9]. However, the role of monocytes and subsets are not understood in childhood obesity. Our hypothesis is that obesity is associated with a predominance of inflammatory monocyte subsets, which could contribute to the pathogenesis of childhood obesity. Clarifying the role of these cells in obesity may provide a lead toward new therapeutic strategies in obesity-association inflammatory disorders.

## Patients, Materials and Methods

### Study Population

The patients who agreed to participate in this study were identified and selected at *Serviço de Medicina Preventiva* (SEMPRE) in Aracaju-Brazil. BMI levels among children and teens need to be expressed relative to other children of the same sex and age. These percentiles are calculated from the Center of Disease Control and Prevention (CDC) growth charts [14], which were based on national survey data collected from 1963–65 to 1988–94. Obesity is defined as a BMI at or above the 95th percentile for children and teens of the same age and sex. We calculated age- and sex-specific body mass index (BMI) percentiles using U.S. national reference data [14]. Eleven children (age 7 to 18 years) were classified with a BMI greater than 95 percentile and included in childhood obesity group (CO). The normal-weight group (NW) was composed by nine children (age 7 to 18 years) with BMI between 10–85 percentile, within the normal weight range. All legal guardians, as well the children, were fully informed about the aim and the protocol of the study and signed an informed consent approved by the Ethical committee of the Federal University of Minas Gerais. Data concerning individuals included in this work are shown in Table 1.

**Table 1:** Clinical parameters.

	Normal-weight (NW)	Childhood obesity (CO)
Total *N*	9	11
Male	4	5
Female	5	6
Age, years	9,33 ± 1,58	9,27 ± 1,27
Height, cm	146,56 ± 13,53	143,18 ± 6,81
Weight, kg	40,06 ± 12,30	55,59 ± 13,28*
BMI, kg/m^2^	17,60 ± 1,55	27,05 ± 4,35*
BMI-per-age, percentile	59,22 ± 26,18	98,00 ± 1,18
Waist circumference, cm	64,11 ± 7,88	82,91 ± 7,44*
Fasting glucose, mg/dL	77,33 ± 12,71	90,45 ± 7,06*
Total Cholesterol, mg/dL	147,11 ± 17,51	181,27 ± 33,26*
LDL, mg/dL	79,22 ± 7,22	97,27 ± 19,98*
HDL, mg/dL	48,33 ± 5,07*	38,73 ± 8,06
Triglycerides, mg/dL	97,33 ± 15,70	174,64 ± 62,58*
AST, mg/dL	12,67 ± 4,03	28,55 ± 11,05*
ALT, mg/dL	16,89 ± 3,44	28,45 ± 11,59*
GGT, mg/dL	15,56 ± 3,47	40,82 ± 21,37*

BMI—Body Mass Index; LDL—Low Densisty Lipoprotein; HDL—High Density Lipoprotein; AST—Aspartate Aminotransferase; ALT—Alanine Aminotransferase; GGT—Gamma Glutamyl Transferase.

Unless otherwise noted, data are presented as mean ± SD.

* Indicates a significant difference by two-tailed *t* test (p<0.05).

### Ethics Statement

Written informed consent was obtained from all legal guardians prior to including the children in the study. Independent of their participation in this study, all individuals enrolled were submitted to a standard screening protocol, follow up and clinical treatment. This study was carried out in full accordance with all International and Brazilian accepted guidelines and was approved by the Ethics Committee at Brazil platform (CAAE– 04065412.6.0000.5526).

### Blood samples

After 12h fasting, 12mL of peripheral blood was collected by venipuncture from each subject using three sterile Vacuntainer^®^ tubes, each with 4mL. Tubes containing ethylenediamine tetraacetic acid (EDTA) as an anticoagulant were used for obtaining whole blood used in flow cytometry assays. Plasma samples were separated from heparinized tubes and serum samples from tubes without anticoagulant; plasma and serum were used for determining the biochemical parameters. Blood samples were collected by a trained nurse at SEMPRE in Aracaju-Brazil.

### Biochemical parameters

Serum lipids and glucose, leptin, adiponectin, aspartate aminotransferase (AST), alanine aminotransferase (ALT), gamma-glutamyltransferase (GGT) and C-reactive protein (CRP) in plasma were measured using different assays. Glucose, triglycerides, total cholesterol and high-density lipoprotein (HDL) were measured by enzymatic-colorimetric assays using a Roche–Hitachi 912 automated analyzer (Hitachi, Nakokojo, Japan). Low-density lipoprotein (LDL) values were calculated by Fridewald’s formula. Leptin, adiponectin, AST, ALT, GGT were determined by ELISA assay (Alexis Biochemical/Vender BioAgency, Sao Paulo, Brazil). CRP was measured by immunoturbidimetry method using the Immage apparatus (Beckman Coulter, USA).

### Flow cytometry analysis of peripheral blood

Peripheral blood from patients was incubated for 4 hours in 5% CO^2^ at 37°C with Brefeldin-A (SIGMA-USA) (10μg/ml). After incubation, 100μL of blood were resuspended and placed in tubes with 2μL of undiluted monoclonal antibodies: anti-CD14 (clone M5E2)–labeled with PerCPCy5.5; anti-CD16 (3G8)–labeled with FITC; anti-CD36 (CB38)–labeled with PE; anti- CD40 (5C3), CD80 (L307.4), CD86 (2331FUN-1), HLA-DR (G46-6), TLR-2 (11G7)–labeled APC or anti-TLR-4 (TF901)–labeled with PECy7 to identify surface molecules. Samples were incubated for 30 minutes at room temperature and in light absence. Next, it were subjected to erythrocyte lysis by incubation with 2mL of a commercial lysing solution (FACS Lysing Solution-BD, USA) for 10 minutes at room temperature. Samples were washed using phosphate-buffered saline (PBS) with 1% bovine serum albumin (BSA), permeabilized using PBS with 0.5% saponin and incubated with 1μL of undiluted monoclonal antibodies anti- IL-1β (AS10), IL-6 (MQ2-6A3), IL-8 (G265-8), IL-10 (JES3-9D7), IL-12 (20C2), TNF-α (6401.1111) and TGF-β (TW4-9E7)–labeled with PE. Samples were washed and fixed with 2% formaldehyde solution, and acquired on flow cytometer FACSCanto™ II (BD Biosciences, Breda, Netherlands). All Antibodies used are from BD Pharmingen™ combined in multicolor panel. Compensations controls for each fluorochrome solely used to set compensation. The analyses were performed using FlowJo 10.2 software (Tree Star, Inc., Ashland, OR, USA). Phenotypic profile of monocytes and their subsets was determined by expression of CD14 and CD16 as previously described [10, 15–17].

### Statistical analysis

Statistical analyses were performed using GraphPad Prism 5.0 software package (San Diego, CA, USA). When data obtained a non-Gaussian distribution, statistical comparisons were carried out using the nonparametric Kruskal-Wallis test, followed by Dunn's multiple test for comparison intragroup of three monocyte subsets. For comparison intergroup of normal-weight or childhood obesity, we used Mann Whitney test for each variable. Clinical data assumed a Gaussian distribution and statistical comparisons were carried out using the *t* student test. Correlation analysis was done using Spearman's correlation coefficient using JMP software (Cary, NC, USA). In all cases, significance was considered at p<0.05.

## Results

### Inflammation and metabolic disorders associated with childhood obesity

Clinical characteristics of CO and NW are presented in Table 1. Anthropometric evaluation showed that CO presents weight (p<0.05), BMI (p<0.001) and waist circumference (p<0.001) were higher than NW. For biochemical parameters, an increase in fasting glucose levels in CO compared to NW (p<0.05) was observed. CO also showed an increase in total cholesterol levels (p<0.05), LDL and triglycerides (p<0.05), characterizing hypertriglyceridemia, and concomitant reduction in HDL-cholesterol levels (p<0.05), featuring a dyslipidemia. Moreover, CO showed abnormal liver function with an increase in the levels of AST, ALT and GGT, compared with NW. GGT levels above the reference range were identified in the CO.

**Fig 1:**
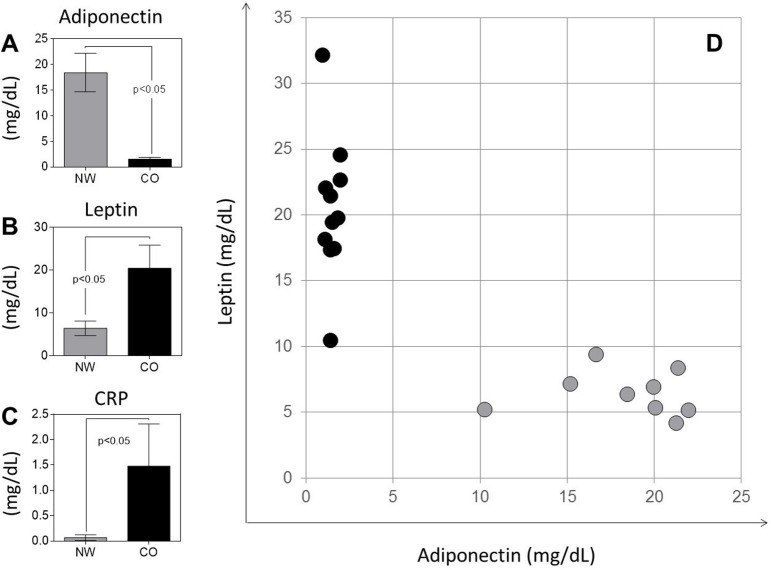
**Plasma levels of adiponectin (A), leptin (B) and CRP (C)** and dispersion graph of adiponectin versus leptin levels (D) are shown. The groups evaluated were normal-weight (NW; n = 9) and childhood obesity (CO; n = 11). Results in panels A, B and C are presented as mean ± SD. Significant differences (p<0.05) in the charts are identified by *p* value and connecting lines.

Adiponectin, leptin and C-reactive protein (CRP) were measured to characterize inflammatory status and adipose tissue physiology (Fig 1). We observed that CO showed a decrease in adiponectin (Fig 1A), and an increase of leptin and CRP (Fig 1B and 1C). Correlation analysis between leptin and adiponectin levels showed a clear segregation between CO and NW (Fig 1D). CO showed elevated levels of leptin combined with decreased levels of adiponectin, while NW group showed opposite profile (Fig 1D).

### CD14^+^CD16^++^ non-classical monocytes is higher in children with obesity

**Fig 2:**
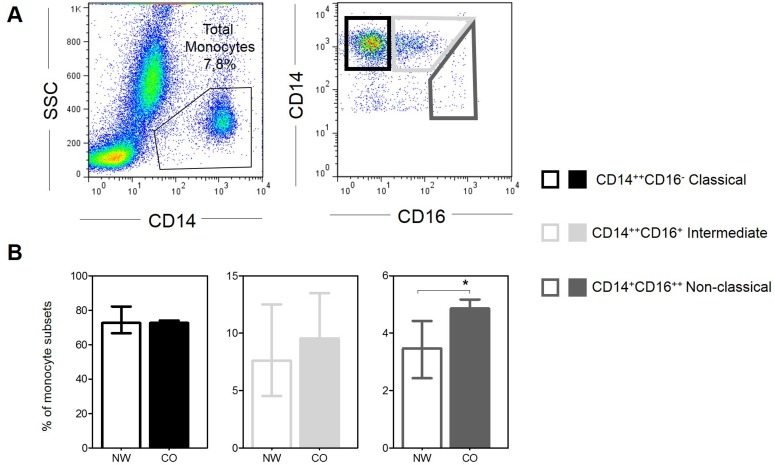
Frequency of monocytes subsets. (A) Representative gates of flow cytometry for classification of subsets. (B) Frequency of each monocyte subsets in normal-weight (NW; n = 9) and childhood obesity (CO; n = 11). Graphs shown median with interquartile range. Significant differences (p<0.05) in the charts are identified by asterisks and connecting lines.

In order to determine if obesity interferes with the frequency of circulating monocyte subsets, we evaluated the frequency of these subpopulations in NW and CO. Fig 2A shows the gating strategy used in these analyses. We did not observe statistical difference in the percent of CD14^++^CD16^-^ classical and CD14^++^CD16^+^ intermediated monocytes (Fig 2B). However, frequency of CD14^+^CD16^++^ non-classical monocytes was significantly increased in CO when compared with NW (Fig 2B).

### Monocyte subsets from CO display altered expression of recognition and activation molecules, as compared to those from NW

**Fig 3:**
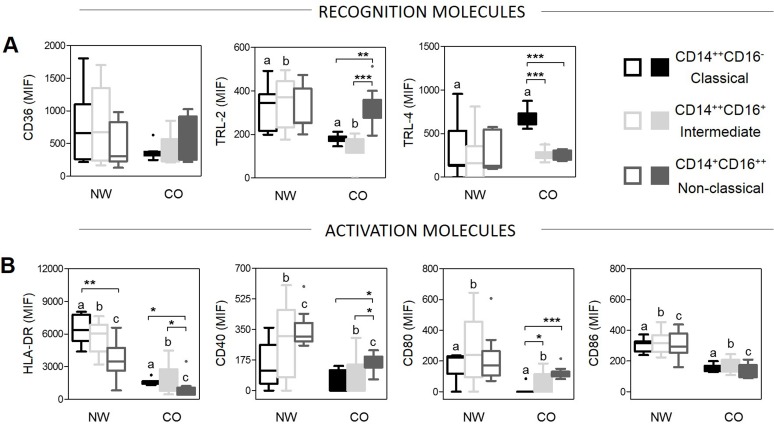
Evaluation of expression of recognition and activation molecules in monocytes and their subsets. (A) Expression of recognition molecules was determined by mean fluorescence intensity (MFI). (B) Expression of activation molecules was determined by mean fluorescence intensity (MFI). The groups evaluated were normal-weight (NW; n = 9) and childhood obesity (CO; n = 11). Significant differences (p<0.05) between monocytes subsets, intragroup, are identified by asterisks and connecting lines. Letters represent statistical differences (p<0.05) between the same subset for comparison intergroup. Graphs shown median with interquartile range.

In order to evaluate whether obesity affected monocytes functions we evaluated the expression of CD36, TRL-2, TRL-4 and HLA-DR, CD40, CD80/86 molecules in NW and CO. Our data showed that CD14^++^CD16^-^ classical and CD14^++^CD16^+^ intermediate monocyte subsets have lower expression of CD36, TRL-2 and HLA-DR in CO compared with NW, which may diminish their capacity to recognize and present antigens (Fig 3). Nevertheless, we observed an increased in expression of TRL-4 in CO compared with NW, especially in CD14^++^CD16^-^ classical monocytes (Fig 3A). Comparing the expression of the different molecules amongst the subsets, we observed that CD14^++^CD16^-^ classical monocytes showed an increase in expression of TRL-4 and CD14^+^CD16^++^ non-classical monocytes showed an increase of TRL-2 compared to others subsets in CO (Fig 3A). These differences were not observed amongst monocyte subsets from NW (Fig 3A).

The activation profile of monocyte subsets was also evaluated and the results demonstrated decreasing expression of CD40, CD80 and CD86 in all monocyte subsets in CO compared to NW (Fig 3B). Within CO, CD14^+^CD16^++^ non-classical and CD14^++^CD16^+^ intermediate presented higher expression of CD80 compared with CD14^++^CD16^-^ classical monocytes (Fig 3B). Moreover, we observed that CD14^++^CD16^-^ classical and CD14^++^CD16^+^ intermediate cells had higher expression of HLA-DR compared to CD14^+^CD16^++^ non-classical monocytes in all groups (Fig 3B).

### Monocyte subsets display lower IL-10 expression during childhood obesity

**Fig 4:**
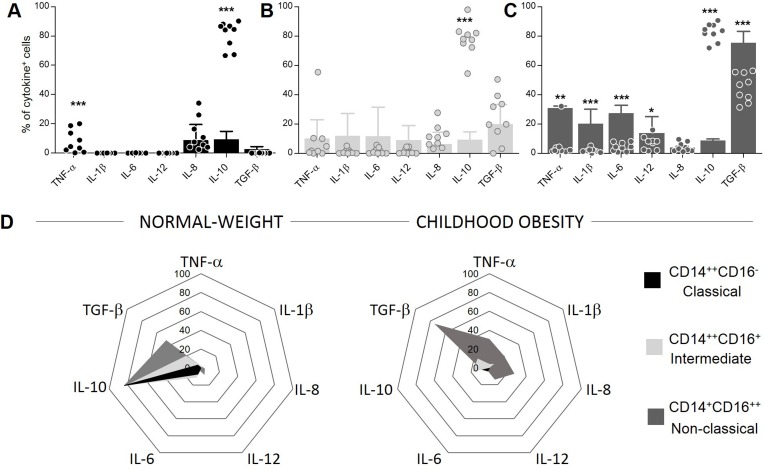
Evaluation of cytokine expression in monocytes and their subsets. (A) Perceptual of classical monocytes expressing each cytokine. (B) Perceptual of intermediate monocytes expressing each cytokine. (C) Perceptual of non-classical monocytes expressing each cytokine. Graphs show median with interquartile range. Significant differences (p<0.05) are identified by asterisks. (D) Radar graph represent perceptual of each monocytes subsets expressing cytokine. The groups evaluated were normal-weight (NW; n = 9; dispersion points) and childhood obesity (CO; n = 11; bars).

Cytokines play an important role in communication of immune system and in inflammatory responses. We observed that CD14^++^CD16^-^ classical monocytes showed lower expression of TNF-α and IL-10 in CO compared to NW (Fig 4A). For CD14^++^CD16^+^ intermediate cells, we observed a lower IL-10 expression in CO compared to NW (Fig 4B). Finally, in CD14^+^CD16^++^ non-classical monocytes, our results showed that CO presented higher expression of inflammatory cytokines TNF-α, IL-1β, IL-6 and IL-12 and a regulatory cytokine TGF-β, but lower IL-10 expression as compared to NW (Fig 4C). We demonstrated using radar graphs (Fig 4D) that all subsets showed decreased IL-10 expression in CO as compared to NW. These data also showed that CD14^+^CD16^++^ non-classical monocytes increased TGF-β expression in CO compared to NW, possibly as an attempt to contain the inflammatory response given the observed decrease in IL-10.

### Childhood obesity change the patterns of immune response in monocyte subsets

**Fig 5:**
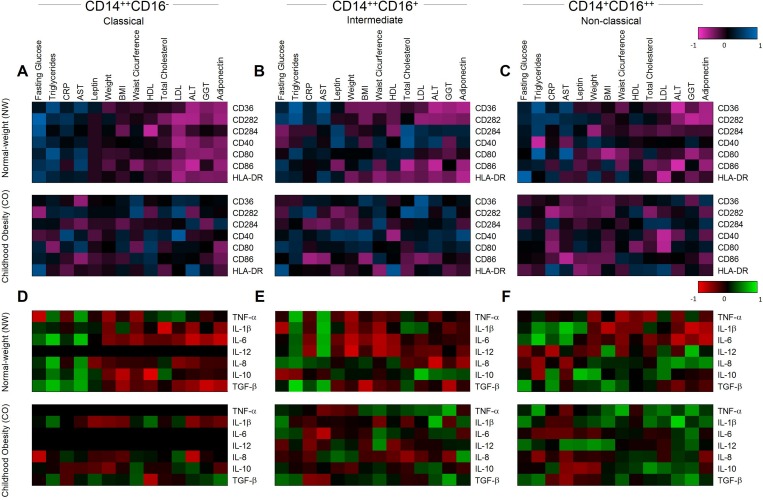
Heatmap for correlations between inflammatory and anti-inflammatory molecules. Representative heatmap of correlation between recognition/activation molecules and clinical parameters for classical (A), intermediate (B) and non-classical monocytes (C). Representative heatmap of correlation between cytokines and clinical parameters for classical (D), intermediate (E) and non-classical monocytes (F). The groups evaluated were normal-weight (NW; n = 9) and childhood obesity (CO; n = 11).

To determine the association between immune response and obesity we performed correlation analysis between clinical and immunological parameters in monocyte subsets. The data was distributed in heatmaps and the groups were hierarchically clustered based on their associated expression. Our data showed visually clear changes in CO compared with NW (Fig 5). CD14^++^CD16^-^ classical monocytes showed positive correlations between fasting glucose, triglycerides, CRP, AST and negative correlations between LDL, ALT, GGT and adiponectin with receptors of recognition/activation (Fig 5A, upper panel) and also for cytokines (Fig 5D, upper panel) in NW. However, clear loss of these associations was observed in CO (Fig 5A and 5D, lower panels). Similarly, CD14^+^CD16^++^ non-classical monocytes showed the same patterns of correlations with recognition/activation (Fig 5C). However, we observed the opposite profile between LDL, ALT, GGT and adiponectin with cytokines in CO compared to NW (Fig 5F). For CD14^++^CD16^+^ intermediate monocytes, we observed a disorganization of correlation profile in CO compared to NW (Fig 5B and 5E) possibly due the transitional behavior of this subset.

**Fig 6:**
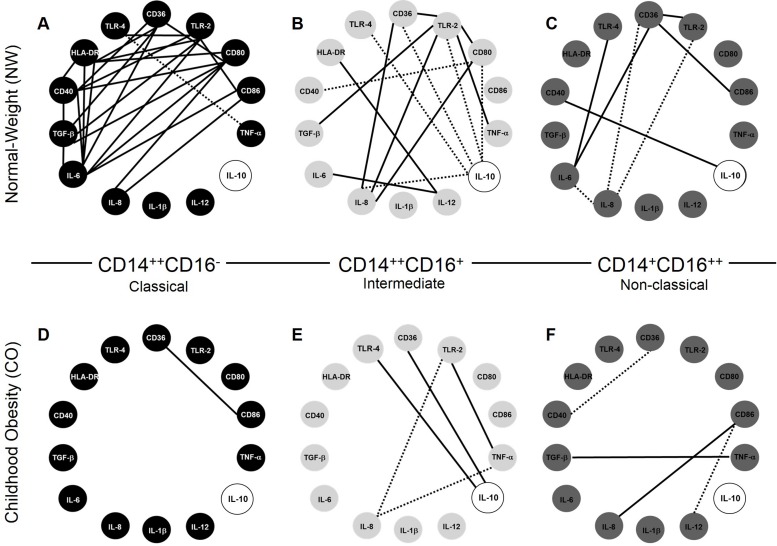
Correlation networks in monocyte subsets. Correlations network in normal-weight group (NW; n = 9) for classical (A), intermediate (B) and non-classical monocytes (C). Correlations network in childhood obesity group (CO; n = 11) for classical (D), intermediate (E) and non-classical monocytes (F). All correlations shown in networks are strong, presents r-squared higher than 0.63, and statistical significance defined by p<0.05. The continuous lines represent positive correlation and the traced lines represent the negative correlation.

Correlation networks between immunological variables expressed by monocyte subsets demonstrated important alterations, especially in IL-10, that showed considerable correlations changes in CO compared to NW (Fig 6). Our data showed that CD14^++^CD16^-^ classical monocytes from CO lose the majority of correlations between recognition, activation and cytokine markers, compared to NW (Fig 6A and 6D). Interestingly, CD14^++^CD16^+^ intermediate monocytes showed the inverse correlation of IL-10 with recognition receptors: positive correlations were observed in NW, and negative correlations in CO (Fig 6B and 6E). In addition, CD14^+^CD16^++^ non-classical monocytes lose all correlations involved recognition receptors CD36, TRL-2 and TRL-4, and appears correlations with activation molecules, mainly CD86 and CD40 in CO compared to NW (Fig 6C and 6F).

## Discussion

Obesity is a condition able to trigger an immune response. The mechanisms that underlie this association are still unclear. However, it is possible that the key regulators of metabolism also play fundamental role in regulating inflammatory responses. Monocytes are more than a transition cell that adapts to a specific tissue setting, but their functions have great influence in physiologic and pathologic conditions. In this work, we observed that patients with childhood obesity have an increase in frequency of CD14^+^CD16^++^ non-classical monocytes. Our data also showed that expression of molecules associated with antigen recognition and presentation are decreased in all monocyte subsets during childhood obesity, suggesting that obesity may affect these processes. In agreement, other studies [18–21] showed that obesity increases susceptibility to infections and leads to disorders in the immune system. Indeed, decrease in expression of antigen recognition and activation molecules may contribute to this status. On the other hand, we showed that the expression of TRL-4 was increased in monocytes from children with obesity, mainly in CD14^++^CD16^-^ classical monocytes. The relationship between nutritional fatty acids, whose circulating levels are often increased in obesity, and TLR4 signaling in adipocytes and macrophages has been previously described [22–24]. This evidence suggests a pathway for macrophage activation that triggers and maintains, at least partially, the inflammation in childhood obesity, resulting particularly in cytokine production.

Our data also showed TNF-α overexpression in CD14^++^CD16^-^ classical and CD14^+^CD16^++^ non-classical monocytes from children with obesity compared to children with normal weight. Despite CD14^+^CD16^++^ non-classical monocytes being described as the origin of M2 macrophage subpopulation with regulatory functions of inflammation in damaged tissues [25–28], we observed higher expression of inflammatory cytokines IL-1β, IL-6, IL-8, and IL-12 by these cells. This suggests that obesity directs this population to a more inflammatory profile. In addition, we demonstrated a decreased in IL-10 expression in childhood obesity, which would be an event that supports expression of inflammatory cytokines, since IL-10 regulates expression of IL-1β, IL-6, IL-8, IL-12 and TNF-α [29, 30] Moreover, this lower expression of IL-10 may be decisive for inducing and/or maintaining the low-grade inflammation status observed in children with obesity. Carolan and colleagues [31] showed that an exacerbated production of inflammatory cytokines and absence/reduction in expression of regulatory cytokines might favor the cytolytic effects of leukocytes, increase the chances of tissue destruction, contributing to the appearance of the metabolic disturbances associated with obesity. We also showed that childhood obesity promoted a substantial loss in the number of significant interactions between different markers, especially with the IL-10 expression. These data reinforce that IL-10 may represent an effective modulatory event on the immune response and its expression directs low-grade inflammation during obesity. Hence, the increase in expression of TGF-β observed in CD14^+^CD16^++^ non-classical monocytes from child with obesity, may be an attempt to control immune response to restore homeostasis, or tissue remodeling and fibrosis [32]. Thus, in childhood obesity, low-grade inflammation seems to change the characteristic of CD14^+^CD16^++^ non-classical monocytes, where TGF-β, rather than IL-10 may assume the regulatory function.

It has been described that the interaction of LDL with CD14^+^CD16^++^ non-classical monocytes may regulate their polarization in M2 macrophage and their capacity to response to different stimulus [33]. Our data showed negative correlation between LDL and most molecules evaluated in children with normal weight, while in children with obesity these correlations were mostly positive. LDL levels may be one of the factors that interferes with CD14^+^CD16^++^ non-classical monocytes physiology. However, considering obesity as a multifactorial disease, where a number of factors contribute synergistically to this disorder, further studies need to be performed to determine LDL involvement. This would have important implications, where controlling LDL levels would be critical to restore immunological homeostasis in obesity.

CD36 is a membrane glycoprotein present in mononuclear phagocytes, adipocytes, hepatocytes and myocytes [34], is also involved in the uptake of oxidized LDL, apoptotic cells and in the modulation of inflammation, atherosclerosis, diabetes, and cardiomyopathy [35]. We observed a positive correlation between CD36 and TLR in CD14^+^CD16^++^ non-classical and CD14^++^CD16^+^ intermediate monocytes from CO. In this context, the expression of CD36/TLR/MyD88 in monocyte-derived macrophages can mediated the production of proinflammatory cytokines (IL-1Ra, IL-1β, IL-6, TNF-α and -β, and IFN-1γ and -β) in response to modified oxidized LDL (oxLDL) [36]. In addition, Tontonoz et al [37] showed that expression of CD36/TLR4 was directly induced by the transcription factor peroxisome proliferator–activated receptor-γ, which has a role in atherogenesis, adipogenesis, and insulin signaling. CD36 can regulate the LPS-induced inflammation process via the NF-κB and c-JNK signaling pathways to modulate downstream cytokine production and interplay with TLR [38]. Then, it is possible that CD36 can act synergistically with TLR in CD14^++^CD16^+^ intermediate and CD14^+^CD16^++^ non-classical monocytes, probably induced by oxLDL, inducing the production of inflammatory cytokines, increasing the low-grade inflammation and the appearance of comorbidities observed during the obesity.

We described correlations between adiponectin with regulatory cytokines IL-10 and TGF-β also between leptin and CPR with inflammatory cytokines TNF-α, IL-1β, IL-6 and IL-12. Indeed, adiponectin, leptin and other mediators have been associated with obesity [39, 40], but their association with the immune response of children with obesity shown in this paper is a new finding. We demonstrated an increase in all clinical parameters evaluated in children with obesity, except for HDL and adiponectin. Thus, our observation of an association between clinical and immunological parameters reinforced the relation of childhood obesity with inflammation and metabolic disorders.

We highlight changes in the CD14^+^CD16^++^ non-classical monocyte subpopulation in children with obesity. These cells seem to contribute to supporting inflammation and loss of regulation in immune response in children with obesity, especially by alteration in IL-10 and TGF-β expression. We believe that these alternations changes the profile of monocytes to be differentiated in deficient macrophages to control inflammation in damaged tissues, contributing to exacerbation of the inflammatory process. Alterations in antigen recognition and presentation molecules may also contribute to the appearance of comorbidities during childhood obesity. Thenceforward, the identification of monocyte subsets as key players in chronic low-grade inflammation opens perspectives for designing new therapeutic strategies in to treat childhood obesity and its consequences in immune response.
